# Perceptions of diet, physical activity, and obesity-related health among black daughter-mother pairs in Soweto, South Africa: a qualitative study

**DOI:** 10.1186/s12889-016-3436-8

**Published:** 2016-08-09

**Authors:** Emily A. Phillips, Dawn L. Comeau, Pedro T. Pisa, Aryeh D. Stein, Shane A. Norris

**Affiliations:** 1 Rollins School of Public Health, Emory University, Atlanta, GA USA; 2 MRC/Wits Developmental Pathways for Health Research Unit, Department of Paediatrics, Faculty of Health Sciences, University of the Witwatersrand, Johannesburg, 2193 South Africa

**Keywords:** Obesity, South Africa, Food, Diet, Physical activity, Body image, Qualitative, Women, Intergenerational

## Abstract

**Background:**

The prevalence of overweight and obesity is on the rise in South Africa, particularly among females living in urban environments. The purpose of this qualitative study was to explore the emic perspectives of black young adult daughter and mother pairs living in Soweto, South Africa on diet, physical activity, and obesity-related health within their social and cultural context.

**Methods:**

Purposeful sampling was used to recruit daughters with a normal body mass index (BMI) who have obese mothers. Individual semi-structured in-depth interviews were conducted with 17 daughters (age 24 years) and 15 of their mothers in Soweto, South Africa. Interview questions related to: a) eating and physical activity behaviors and perceptions, b) perceptions of social and community level factors, c) cultural beliefs about diet and body image, and d) intergenerational relationships. Data were analyzed using four-phases of thematic analysis and the constant comparison approach.

**Results:**

Daughters and mothers had similar ideas of the definition of healthy food and the importance of eating healthy, but mothers were more likely to report eating healthy because of their age, adverse health experiences, and a desire to live longer. Daughters and mothers engaged in physical activity for reasons related to weight maintenance and feeling better, but mothers reported being more likely to start exercising as a result of a health concern. Daughters and mothers had comparable views of what makes a person healthy. Daughters and mothers relied on each other for food purchasing and food preparation.

**Conclusion:**

Daughters and mothers shared some similar perceptions of diet, physical activity, and health that were rooted in their daily life in Soweto. However, mothers generally reported being more likely to exhibit healthy eating and physical activity behaviors despite being obese. The mothers may have adopted these perceptions and behaviors later in life linked to ageing and ill-health. It is possible that through exposure, their daughters have assimilated these perceptions earlier in childhood or adolescence. It is important to focus health promotion efforts around preventing the otherwise expected increase of obesity among the young adult generation.

## Background

An estimated 13 % of the world’s adult population is considered obese (body mass index (BMI) >30 kg/m^2^) and an additional 39 % is overweight (30 ≥ BMI >25 kg/m^2^) [1, 2]. Obesity, an independent risk factor for many non-communicable diseases [3, 4], has become a major public health issue in developing countries [3, 5]. Middle income countries, such as South Africa, are characterized by an epidemiological and nutrition transition that is accompanied by increased prevalence of non-communicable diseases, undernutrition, infectious diseases, and the human immunodeficiency virus (HIV)/AIDS pandemic [6–10]. South Africa, the second largest economy in Africa, has the highest obesity prevalence rates on the continent [11]. Results from the 2012 South African National Health and Examination Survey estimated that 25 % of adult South Africans are obese and a further 22.5 % are overweight [12]. Obesity is disproportionately higher among South African females, with a prevalence of 39.2 % among females as opposed to 10.6 % among males [12, 13]. South African females experience an increased risk of obesity with age and by living in an urban area [9, 12–19]. While obesity is a complicated issue, lifestyle, cultural, urbanization, and environmental factors are important determinants of increased obesity levels in South Africa [14, 20].

In South Africa, black females are at highest risk of overweight and obesity compared to other ethnicities [14, 20]. Other than older age and living in an urban area, important factors associated with obesity among females in South Africa include having at least one overweight parent, marital status, physical inactivity, high birth weight, and diets higher in sugar, fats and refined carbohydrates and lower in vegetable and fibrous fruit intake [13, 20–24]. Cultural and traditional values also influence the prevalence of larger body size among black South African females. Larger body size is generally viewed as a sign of beauty, health, prosperity, and the absence of disease or illness including HIV/AIDS [13].

Research findings suggest that there is a correlation between maternal and child body mass index, including that of adult children [25–27]. Specific to South Africa, strong mother-daughter relationships among black South Africans have been found for a number of body size characteristics, including perceptions of ideal body sizes [28]. Given that South African females are more likely to become obese if they have at least one overweight parent, the mother-child relationship is an important factor to consider when aiming to understand obesity.

Currently, there is a lack of qualitative literature that explores the intergenerational dynamic and how it relates to food, physical activity, and obesity-related health in urban South Africa. The purpose of this qualitative study was to explore the emic perspectives of black young adult daughter and mother pairs living in Soweto, South Africa on diet, physical activity, and obesity-related health within their social and cultural context. Since South African females are more likely to become obese if they have at least one overweight parent [20–23], this study aimed to primarily understand daughter-mother relationships that deviated from the norm. This approach may allow insight into how daughters who were prone to overweight and obesity maintained a normal BMI into early adulthood [29].

## Methods

### Eligibility and participant recruitment

**Fig. 1:**
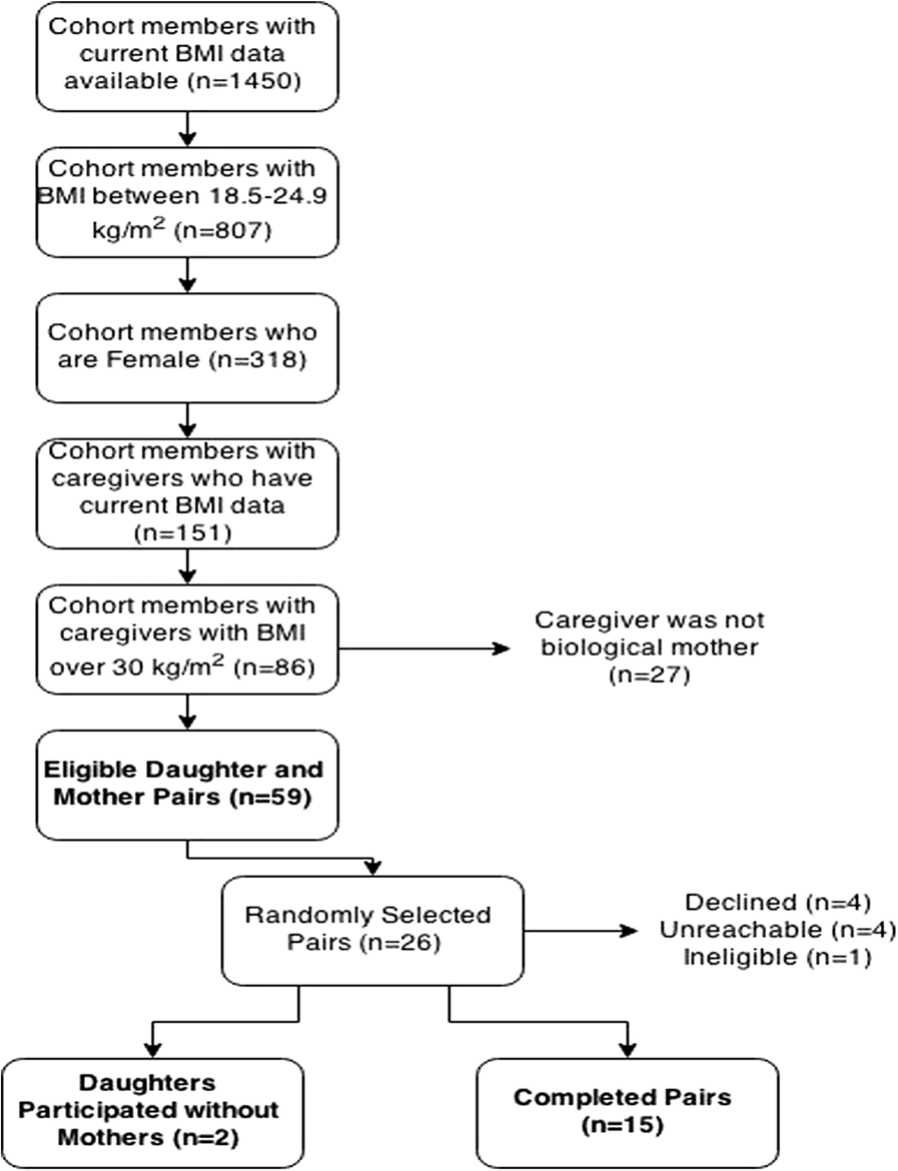
Purposive sampling and recruitment

Birth to Twenty Plus (Bt20) is a longitudinal study in Johannesburg-Soweto, South Africa that includes singleton children (*n* = 3273) born within a seven-week period in 1990 [30]. Young adult female members of the birth cohort and their mothers were purposefully sampled. Eligibility criteria included: (a) young adult women with a BMI in the normal range (between 18.5 and 24.9 kg/m^2^), (b) who lived in Soweto, and (c) had a biological mother who was obese (BMI > 30 kg/m^2^). Figure 1 demonstrates how Bt20 cohort members were purposefully recruited for study participation.

Eligible participants were assigned a unique study identification number and pairs were randomly selected to participate [31]. Randomly-selected daughter-mother pairs were contacted via telephone by a Bt20 research assistant and were invited to participate in the study. Both the daughter and mother had to be willing to participate in order to be involved.

### Interview guide

Two semi-structured interview guides were developed; one for daughters and one for mothers. Interview questions spanned the following domains: a) eating and physical activity behaviors and perceptions, b) perceptions of social and community level factors, c) cultural beliefs about diet and body image, and d) intergenerational relationships. A core set of questions addressing the four domains were used in both interview guides, and several additional questions were tailored to the mother or daughter about their experiences with each other. A focus group with Bt20 research staff reviewed the interview guides and provided feedback on the language of interview questions and cultural appropriateness of topics and context. Pilot interviews were conducted with two pairs, and subsequent changes to the interview guides were made to improve the natural flow of the conversation and to allow for more probing questions [32]. As is convention in qualitative methods, interview guides were adjusted throughout data collection to incorporate questions about emerging topics [33, 34]. There were three iterations of each interview guide.

### Data collection

Semi-structured, in-depth interviews were conducted at Chris Hani Baragwanath Hospital in Soweto, except for one that was conducted at the participant’s home because of the participant’s preferences. All data collection for Bt20 has occurred at Chris Hani Baragwanath Hospital, so participants were familiar with the interview setting. Interviews were conducted in private research rooms by a female graduate researcher trained in qualitative research methods. All interviews were conducted in English except for one that was conducted in Sesotho. A bilingual research assistant provided simultaneous translation for the English-speaking interviewer. Mothers and daughters were generally interviewed on the same day.

Written informed consent was collected from each participant before the start of the interview. Prior to the start of the interview, participants were asked to provide a pseudonym in order to protect identity. A demographic questionnaire was administered before the interview. Demographic data variables included date of birth, marital status, occupation status, education, parental status, and home description.

Participants were reimbursed for travel expenses and were provided refreshments. Interviews ranged from 22 to 80 min. Data were collected in June-July, 2014. The study received approval by the University of the Witwatersrand Ethics Committee [M140479] and Emory University Institutional Review Board [IRB00073854].

### Data analysis

Interviews were audio-recorded, transcribed verbatim, and de-identified. All transcripts were entered into MaxQDA 11, a qualitative software for data management, coding and analysis. We reviewed a subsample of transcripts and generated a list of inductive and deductive codes to capture ideas present in the text. The list of codes was discussed with qualitative research professors at Emory University and graduate student researchers familiar with the study and refined according to feedback. The final codebook included codes, definitions and example quotes. Deductive codes included themes based on the interview guides and literature review and the inductive codes captured new themes that emerged throughout data collection. Throughout the coding process, the study aims were revisited with particular attention focused on the daughter-mother dyad.

Data analysis occurred in four consecutive phases:

1. Phase One: Case-based analysis within the subsample of daughters.
  We examined the data collected from daughters about eating and physical activity. Data were entered into a matrix and we compared the knowledge, perceptions, and behaviors of healthy eating and physical activity throughout each participants’ interview. This process resulted in thick, rich description about each young woman’s experience with eating and physical activity.
2. Phase Two: Thematic analysis across daughters and mothers.
  We examined the data by theme across the interviews. We used the constant comparison method to note similarities and differences between the daughters and the mothers. We made note of instances in which the participant’s experiences deviated from the typical findings and the context of these situations. If needed, we reread the original transcripts to broaden our understanding of the women’s discussions about eating, physical activity, and health.
3. Phase Three: Case-based analysis within daughter-mother pairs.
  In order to fully explore the relationship between daughters and mothers and their perceptions of diet, physical activity, health, and body image, we created an additional matrix to compare passages of text in which daughters spoke about their mother, and mothers spoke about their daughters. Each daughter-mother pair was analyzed as a case and we highlighted key thematic findings.
4. Phase Four: Thematic analysis across daughter-mother pairs.
  A focused analysis was conducted to compare and contrast daughter-mother pair’s experiences. Key themes centered on cultural perceptions of health and body size and possible explanations for differences in BMI.

## Results

### Participants

A total of 32 women participated in this study: 17 daughters and 15 mothers. Data saturation was reached after 32 total interviews. Two participants (daughters) were excluded from Phase 3 and Phase 4 analysis. The mean age of the daughters was 24.2 years (SD = 0.04), and the average age of mothers was 53.0 years (SD = 4.9). Daughter’s mean BMI was 21.6 kg/m^2^ (SD = 1.8), and mother’s average BMI was 36.1 kg/m^2^ (SD = 3.9) (Table 1).

**Table 1:** Demographic descriptive statistics of participants

Demographic variable	Young adults	Mothers
	N	N
Number of participants	17	15
Age (years)	24.2 (SD = 0.04)	53.0 (SD = 4.9)
BMI (kg/m^2^)	21.6 (SD = 1.8)^a^	36.1 (SD = 3.9)^b^
Education level		
Grade 6	0	2
Grade 8	0	1
Grade 10	0	3
Grade 11 (Matric for mothers)	1	4
Grade 12 (Matric for young adults)	6	2
Certificate/Diploma	4	2
Bachelors	6	1
Marital status		
Single	8	3
Committed Relationship	7	1
Married	2	8
Divorced	0	1
Widow	0	1
Employment status		
Unemployed	5	6
Part Time	2	1
Full Time	10	8
Maternal status		
Yes	6	N/A
No	10	N/A
Pregnant	1	N/A

^a^Average age at BMI Measurement (years): 22.7 (SD = 0.53)

^b^Average age at BMI Measurement (years): 51.2 (SD = 4.8)

### Daughters’ perceptions on diet, physical activity, and obesity-related health

Daughters typically defined healthy foods as fruits, “veggies”, drinking water, cereals, boiled foods, not cooking with a lot of oils, not cooking with spices, and not eating take-aways and fast foods often. Many of the daughters generally thought that eating healthy is a major part of a healthy lifestyle. For some this translated into their own eating habits, and others it did not. Several daughters did not view healthy eating as a priority, despite what they may know about the benefits. For instance, when talking about healthy eating, Bontle said that she never thinks about healthy eating.

I think if you really have to, then you should eat healthy foods, you know? When I get the chance, honestly, I do eat healthy but I don’t even think about healthy eating… It’s not that I do not have the knowledge about healthy eating. It’s there, I know I should eat healthy but yeah, I’ll do it sometime in life…I’ll deal with whatever repercussions of eating junk now later in life, whenever – if I have to anyway.

However, one of the most discussed “healthy” food behaviors among daughters was in relation to food portion size. Most of the daughters said that they preferred to eat smaller or normal size meals. For example, when describing meals with her family, Hope mentioned, “But my sister and I don’t like eating a lot at night so it’ll be just two spoons of rice, for instance, and one spoon of meat, and on a Sunday, it would be vegetables.”

The majority of daughters were not intentionally exercising around the time of the interviews, but most had been regularly active in the past or tried exercising before. Several daughters played popular sports when they were in primary and high school, including netball and tennis. Daughters who exercised reported that they were physically active because they wanted their bodies to feel better and it helped maintain a healthy weight. Daughters who did not exercise knew the benefits of exercise, but either had personal reasons not to exercise or faced barriers to exercise. Some personal reasons not to exercise included having other priorities, preference for sedentary activities, or not enjoying physical activity. A few of the barriers to exercise included a lack of time, a lack of money, lack of access to sports teams, or feeling tired.

Daughters had a broad range of knowledge on the consequences of being unhealthy and what it meant for someone to be healthy. The three most common types of unhealthy outcomes discussed were heart problems and cardiovascular complications, issues related to pain and physiological functioning, and higher exposure to illnesses and sicknesses. When asked to talk about what it meant for someone to be healthy, most daughters mentioned someone who exercises, eats well, and looks out for and takes care of themselves. When discussing how they learned their interpretation of healthy eating, daughters mentioned primary and secondary school (*n* = 7), television (*n* = 4), other people, including friends, siblings, or grandmother (*n* = 4), clinics (*n* = 3), internet (*n* = 2), the work place (*n* = 1), and the mall (*n* = 1). Daughters did not identify mothers as a primary source of health information. Some of the daughters posited that people who have had some adverse experience related to health are more likely to be health conscious and to eat healthy. These experiences serve as a motivation to try to be more health conscious than normal. For instance, Lizzy described,

Say for instance if maybe somebody were to see somebody die of diabetes, then you’re forced to eat healthy because you don’t want to go through that route…So it’s an experience that will make you eat healthy in Soweto.

Most daughters thought that a woman of the same age must not be too thin or too fat, and that there can be negative health consequences for being too skinny. Further, a woman’s weight can be an indicator to others about her age and her life experience. For instance, a woman who gains weight may be recently married, is happier with her life, and is growing up. When describing an ideal body size for woman in their age group, daughters preferred to be around sizes 30–34 (approximately sizes 2–10 in the United States).

Daughters offered several thoughts and recommendations about how women in their communities could be healthier. These suggestions can be characterized into four general areas: healthy eating, physical activity, individual motivations and mindsets, and society. Many daughters thought that people could start to eat healthy if they incorporated healthy food into their diets incrementally. Even though Lebo admits that it is difficult for her to eat healthy, she says,

I will just say they must try, bit by bit, because you cannot just jump at that thing [eating healthy] - it will be very difficult. That is what I am trying now, it is very difficult but bit by bit I will get there…there is a lot of things that happen in a woman's body, so it is very important for us to be healthy.

Similarly, daughters offered that women could begin to exercise if they focused on making small behavior changes first. For instance, Superntha said, “I could say they could start with walking maybe. And then just take it slowly from there, you know. Maybe start jogging. Then maybe as time goes that’s when they can start considering…doing stomach crunches, maybe sit-ups and all that.” Daughters discussed the importance of individuals having personal motivation to be healthy and not being stressed about body weight. For Anne, she thought that in order to lead a healthy lifestyle, “It shouldn’t be like an everyday thing and stressing yourself on how to keep your body healthy. But it should be in your head that you want to be healthy.” Beyond the individual level, some daughters thought that there was a critical need to help women maintain healthy lifestyles during the time period between high school and adulthood. Further, daughters speculated that there are several opportunities for community health interventions (Table 2).

**Table 2:** Daughters’ suggestions for healthy change

Topic	Suggestions
Eating healthy	• Increase consumption of: • water, fruits and vegetables, fiber, foods from across the food chain • Reduce consumption of: • fizzy and cold drinks, oily foods, salt, sugar, animal based foods, dairy products, sweets, and junk, reuse of old cooking oil • Begin to eat healthy bit by bit • Vegetables need to be prepared in an exciting, tasty way
Physical activity	• Start small and work your way up to more difficult activities • Start with walking, then jogging, then calisthenics • Jog before you join the gym so you don’t get tired and waste money • It’s the little things • Walk instead of drive, use the stairs instead of the lift • Do work around the house that makes you sweat • Overcoming issues around safety: • Walk with a friend/in a group • Avoid traffic by jogging in the streets early after morning rush • Walk/jog when it is not dark, and not too late into the evening • Even though there may be crime, you are safe if you are from there because people will look out for you and will notice if there is something suspicious going on • Let someone know where you are going and what you are doing • Do not walk the same time every day to avoid creating a pattern
Individual motivations/Mindsets	• People need an individual motivating factor to help them be healthy. If they can’t see what the benefit is for them individually, it won’t matter. • “You can take a horse to the river, but you can’t make him drink” • Don’t stress about eating healthy – obsessing about weight makes you gain weight. Just eat normally. • People need self-discipline to both eat healthy and exercise • Need to do things now in order to care for yourself in the future. Prevent problems before they happen. • Some people can’t help how they eat, and it’s not sufficient to just say ‘be strict.’ They need social support and more direction with their diet.
Society	• Government Intervention • Education and awareness: need to show people how they can be healthy when they don’t have money, not why it is important • Address unemployment and access-related issues before addressing types of foods people should eat • Support community initiatives that turn unused space into gardens, etc. • Make junk food less available • Programs about healthy lifestyles directed at children need to be exciting • Target children when trying to change mindsets around health • The time between high school and adulthood is the critical time when women don’t have access to physical activity, and lose the habit

### Daughters as told by their mothers

Daughters’ reported eating behaviors was supported by the interviewer’s discussions with the mothers. The majority of mothers indicated that their daughters generally “don’t eat that much.” Mothers often said that despite the fact that their daughters ate take-aways and fast food, and fried and starchy foods at home, daughters almost all preferred to eat small amounts or regular size portions. Hope’s mother, Thato, described her daughter’s eating habits, “She [Hope] doesn’t eat a lot and she doesn’t eat junk food.”

Now that their daughters were 24 years old, most mothers discussed letting their daughters make their own food decisions without interfering. Likewise, daughters reported more control over what they eat compared to when they were growing up. Despite daughters feeling like they had more control over what they eat, most daughters and mothers were still dependent on each other for food. This interconnection was determined by whether or not the daughter was employed. Most daughters who were unemployed or who worked part time were one of the primary cooks of their family’s house. Therefore, their mothers ate whatever their daughters cooked. However, even if their daughters were one of the primary cooks, mothers bought the majority of the groceries for the family. As a result, the mother determined what food is purchased and what ingredients the daughter had available to cook. For example, Nozuko (mother) purchased food for her house, and her daughter Nthabiseng cooked most often during the week because her mother had a full time job. When discussing her daughter’s cooking, Nozuko said, “My daughter cooks, of course, rice… She’s the kind of lady who does not cook vegetables, so I make it easy for her by buying frozen vegetables. Then she just takes two minutes of her time.” In households where daughters were employed, their mothers were generally the primary cook. Daughters were then dependent on their mother’s cooking for most meals throughout the day, except when they bought take-aways at work for lunch. For instance, Smangele (mother) typically cooks for Tozi’s (daughter) household, and Tozi talked about her typical eating patterns during the work week, “Monday to Thursday I bring along my lunchbox… Friday I don’t take lunch boxes. I buy something at work then I eat it.” Employed daughters were likely to contribute to the household finances for food and groceries. In instances where mothers had to change their diet due to health problems or concerns, their daughters only sometimes changed what they ate too.

Many of the mothers in this study were motivated to raise their daughters differently than how they were raised. Mothers wanted their daughters to grow up “better” than they did, so they tried to make their daughters happy by providing different foods for them. For some mothers, the desire to provide their daughters with a better upbringing than their own meant buying more fast foods, like for Beauty (mother):

When I was growing up…we were poor. Then you didn’t have… money to go for take-aways and everything, you only come home and eat home…And another thing that is killing us people here, because I grow up hard, I didn’t want my daughter to grow up like that. Then I will just spend everything, doing everything… When my child was 7, my child must go to McDonald every week. I grow up, I didn’t go to McDonald. That’s the thing that is happening.

Other mothers reported providing healthier foods to their daughters than the foods they ate growing up. For example, Sybil (mother) explained:

I’ve taught myself [to eat healthy] and I’ve told myself that I will never bring my children up the way I was brought up. There must be a change. There must be a difference…I’m trying to say, my mother, she [could not] afford all those fancy food[s]. Butter was a luxury to us. A fruit…I would even forget that there was an apple because my mother [could not] afford to buy it for us. We would eat pap [maize porridge] and acha [African grain] as a meal when we grew up. But now with my children, it would be a choice if they want to eat that.

Almost all mothers discussed that at some point, they instructed their daughters on how they should eat and manage their weight through increased or decreased physical activity. Some mothers told their daughters that it is important to always work around the house as a part of their domestic responsibilities, which many of the participants viewed as a form of exercise. When talking about her family’s views on exercise, Superntha (daughter) laughed and said,

With my mom, you always have to be doing something around the house…Not just sit down and just do nothing. Yeah she doesn’t encourage just sitting, just sitting like that the whole day, no. You have to do something just to keep your body moving.

Other mothers mentioned discouraging their daughters from participating in physical activity because it interferes with more pressing priorities. For example, Khosi, Chelly’s mother, supported Chelly’s soccer activity as a child but did not want her to continue playing as an adult. She explained, “I do talk to her about soccer…because I don’t like soccer. She’s really old now for soccer. She must focus on something else.” Further, Mokgadi told her daughter Dimakatso not to join the gym because the gym was a waste of money and she would not be likely to go every day.

### Mothers’ perceptions on diet, physical activity, and obesity-related health

Mothers agreed with their daughters on typical types of healthy foods and that eating healthy is a major part of a healthy lifestyle. Unlike their daughters, mothers were more likely to think about the health of their diets. Furthermore, many mothers discussed changing their diets to accommodate either personal health concerns or the needs of family members with health issues. Some of the daughters agreed that mothers thought more about healthy eating than young people because they are older and need to eat healthy to live longer. For instance, Anne (daughter) described her mother, Tammy,

Yo, my mom is a health freak. She’s like, she doesn’t eat…normal fish oil thingies. She’s into your olive oils. The butter she buys is the one that says your heart will be healthy or something. The chicken she buys has no skin. I think it [her mom being a health freak] goes back to that age thing. She’s getting older, she wants to be healthier, she wants to live longer.

Mothers had similar reasons to not be physically active and experienced similar barriers to exercise as their daughters. However, mothers who reported that they exercised were more likely to begin exercising as a result of a health issue. For example, Nozuko (mother) relayed her story,

Exercise is very important. I give an example…My blood pressures always high because I always live with peoples’ problems. The doctor says to me, ‘You know, I can’t bring down your blood pressure. I don’t know what to do anymore. Do you exercise?’ And guess what, we do have a gym and I hardly went. In January, I said to myself, ‘This year I’m going to go to gym at least 3 times a week’ …So from January I started to go to gym 3 times a week. End of January, I went to the doctor and he was shocked. It was gone. So exercise works.

The daughter’s perception that people who have had some adverse experience related to health are more likely to be health conscious and to eat healthy was supported by a number of the mother’s personal experiences. For instance, Lucia (mother) relayed that she had a stroke three years before the interview. She explained,

I feel healthy, very healthy and I think the healthy food keep you strong and keep you healthy. Because if it wasn’t through healthy food, I maybe wasn’t going to survive the mild stroke that I have. I’m looking very, very much on my health so that I can live longer.

Mothers shared similar views as their daughters on women’s body size and weight. However, there was a contrast regarding the definition of the ideal body size for women in their age group. When describing an ideal body size for a woman in their age group, daughters preferred to be around sizes 30–34 (approximately sizes 2–10 in the United States), whereas mothers preferred to be around size 38–40 (approximately sizes 14–18 in the United States).

## Discussion

The purpose of this qualitative study was to explore the emic perspectives of black young adult daughter and mother pairs living in Soweto, South Africa on diet, physical activity, and obesity-related health within their social and cultural context. Since South African females are more likely to become obese if they have at least one overweight parent [20–23], this study aimed to primarily understand daughter-mother relationships that deviated from the norm.

Consistent with previous research with non-deviant samples, daughters did not necessarily report healthy eating and physical activity behaviors [35]. Even though daughters and mothers generally had the same definition of healthy foods and the importance of a healthy lifestyle, healthy eating seemed to be more important for mothers. Additionally, mothers were more likely to initiate exercise to address a health concern than their daughters. This may seem contradictory given that the mothers had a much higher BMI than their daughters. It is possible that mothers primarily described present behaviors and perceptions around food, physical activity and health, and their current behaviors and perceptions may not reflect what contributed to their BMI status. Furthermore, both mothers and daughters supported the concept that people in Soweto are more likely to begin to eat healthy if they or a loved one experiences a negative health outcome. Hence, mothers may make dietary and lifestyle changes as a treatment mechanism as result of a negative health outcome. Interestingly, this perception did not encourage the daughters to eat healthier foods or increase physical activity. This supports the premise that it takes more than increasing knowledge to encourage healthy behavior [36].

Daughters and mothers agreed that women should neither be too thin nor have too much body weight. Additionally, they perceived that a woman’s body weight could be an indicator for where she is in her life; this is consistent with prior research in South Africa [28]. Despite these similarities, daughters perceived an ideal body size to be smaller than that of mothers, highlighting a key intergenerational difference. The data from this study does not indicate whether the participants’ BMI status or age explain this difference. However, this finding supports other research which suggests that cultures change and adapt a thinner ideal as they westernize and modernize [37].

Some mothers highlighted that they were motivated to raise their daughters with better circumstances than what they had growing up, which often translated to the types of food they provided for their daughters. However, these motivations were not necessarily driven by health or the desire to prevent negative health outcomes. Since all of the daughters were born in 1990, they were all raised during the beginning of the post-apartheid era. This was a unique time in South African history, which may have played a role in the types of food that was available during the daughters’ childhood compared to what was available for their mothers. Interestingly, young adult daughters and their mothers still relied on each other for food despite their perceived level of autonomy and control over their diets. As such, the interactions between daughters and mothers present food behaviors do not clearly indicate how daughters maintain a normal BMI despite expectations of previous research. These data illustrate that the household is a key environment to consider along with socioeconomic status when trying to understand the cultural context of obesity and development future health intervention and prevention initiatives.

The findings did not provide clear evidence for how daughters who were prone to overweight and obesity maintained a normal BMI into early adulthood. However, this study provides a unique exploration of an intergenerational dynamic that deviates from expectations of previous research.

### Implications of findings to community interventions

Findings from this qualitative study can be used to inform community-based health promotion activities in South Africa or similar settings, and all efforts should be monitored and evaluated for appropriateness and effectiveness. Most participants had a fair amount of knowledge on what it meant to eat healthy and exercise. Health education efforts should build on this knowledge and strengthen women’s understanding of healthy eating and physical activity and how they can achieve healthier lifestyles in Soweto. Skill-based approaches to health education could include how to eat healthy as a family with limited financial resources, how to prepare healthy food that is still typical of common South African foods, and how to cook appetizing food with healthier and less oils. Health interventions could be guided by promoting the South African food-based dietary guidelines on healthy eating, which were updated in 2013 [38, 39].

There are also ways to incorporate health promotion efforts at the organizational and community level. Since mothers and sometimes daughters buy groceries for the whole family, health promotion efforts at major groceries may be helpful in encouraging healthy food purchasing. As a result, healthier food may be more readily available at the home for whenever it is time to cook. In addition, it is important to address the perception that females typically stop engaging in sports and physical activity around adolescence. Since post-high school is a critical time when physical activity tends to decrease among females, it would be valuable to increase support for physical activity opportunities during this transitional time period. The formation of more adult netball teams would be beneficial for women who played in school and wanted to continue playing afterwards. Another possible intervention could be income-based gym and fitness club memberships to encourage people with fewer resources to join and exercise. Finally, an additional avenue for public health efforts is at local schools. For instance, the Life Orientation curriculum implementation for Grade 12 could be adjusted to support one of its objectives: “long-term engagement in traditional and/or non-traditional sport or playground and/or community and/or indigenous games or relaxation and recreational activities” [40]. Research indicates that there is room for improvement in the Life Orientation curriculum, so this may be a targeted way to approach improvements [41].

Local and national decision makers can support community-based initiatives that aim to promote health. These include repurposing empty space for community-based gardens or park development. However, it is critical that these initiatives are community-driven so that they are sustainable and do not reflect an imposition on personal freedom due to the lived memory of the apartheid government. While conducting health promotion activities, it is important not to promote a culture of obsession with being healthy and thin, or one that shames individuals who are overweight or obese. This has been seen in other contexts and is not beneficial to overall mental and physical health [42–45]. Mental health needs to be a priority along with physical and community-level health.

### Strengths and limitations

To our knowledge, this is the first study to date that involves interviewing mother-daughter dyads about intergenerational relationships and influence on eating and physical activity. This provides important cultural context to the implementation of public health interventions. However, this study focused on mothers and daughters when the daughters were young adults. Future studies should continue to explore the influence of mother-daughter relationships on eating, physical activity, and health over the life course.

During data collection, mothers and daughters generally arrived to the study site together. The participants were interviewed separately in a private office, however it is possible that they felt limited confidentiality with their family member waiting outside of the office. During data analysis, the majority of the coding was completed by the lead researcher, which limits assessment of inter-coder reliability. However, a second researcher who was familiar with the study topics and objectives coded a subset of transcripts to confirm the definition and application of codes. The two researchers discussed the discrepancies in coding and adjusted code definitions as needed.

## Conclusion

Despite differences in BMI status, daughters with a normal BMI and mothers with an obese BMI shared some similar knowledge and perceptions of diet, physical activity, and obesity-related health that were rooted in their daily life in Soweto. However, mothers generally reported being more likely to exhibit healthy eating and physical activity behaviors despite being obese. The mothers may have adopted these perceptions and behaviors later in life linked to ageing and ill-health. Further, daughters and mothers still relied on each other for food purchasing and food preparation in adulthood. The findings did not provide clear evidence for how daughters who were prone to overweight and obesity maintained a normal BMI into early adulthood. However, this study provides a unique exploration of an intergenerational dynamic that deviates from expectations of previous research. Health promotion activities can incorporate healthy perceptions of food, physical activity, and health among these two generations of women to address the prevalence of obesity in South Africa.

## Abbreviations

BMI, Body Mass Index; Bt20, Birth to Twenty
